# The effect of two types of pyramidal and reverse pyramidal loading on spine and pelvis coordination variability during deadlift

**DOI:** 10.1186/s12891-026-10111-9

**Published:** 2026-06-20

**Authors:** Mostafa Sajedi Nia, Mehdi Khaleghi Tazji, Ali Abbasi

**Affiliations:** 1 Department of Biomechanics and Sports Injuries, Faculty of Physical Education and Sports Sciences, Kharazmi University, Tehran, Iran; 2 Department of Sport Sciences, Faculty of Education and Psychology, Shiraz University, Shiraz, Iran

**Keywords:** Deadlift, Pyramidal loading, Reverse pyramidal loading, Spine-pelvis coordination, Vector coding

## Abstract

**Purpose:**

To investigate the effects of pyramidal and reverse pyramidal loading on spine and pelvis coordination patterns and their variability during deadlift.

**Methods:**

Twelve trained male powerlifters performed deadlifts under two loading protocols: pyramidal (50%, 70%, 90% of 10RM) and reverse pyramidal (same loads in descending order). Segmental coordination (PL–LB, LB–LT, LT–UT) was analyzed using 3D motion capture and a modified vector coding method across concentric and eccentric phases. Coordination pattern frequencies and variability were statistically assessed using repeated-measures ANOVA and Statistical Parametric Mapping (SPM).

**Results:**

In-phase coordination patterns were dominant in the PL–LB and LB–LT couplings across all intensities. However, pyramidal loading led to increased distal dominance and reduced proximal contribution, particularly at 90% intensity. Significant differences in coordination patterns were observed between the two loading strategies, while coordination variability did not differ significantly.

**Conclusion:**

Pyramidal loading imposes greater neuromechanical demands on trunk segments, increasing the risk of coordination breakdown and potential injury in distal spinal regions. Reverse pyramidal loading may offer a safer alternative for high-load resistance training. Further research is warranted under maximal or fatigue-inducing conditions.

## Introduction

Resistance training is a fundamental component of athletic conditioning, enhancing muscular strength and improving whole-body biomechanics. Among various resistance exercises, the deadlift is a compound movement that engages multiple joints and large muscle groups simultaneously, making it highly effective for developing strength in both the upper and lower body. In addition to promoting muscular hypertrophy, it enhances physical performance, improves core stability, and reduces the risk of injury [1, 2]. Deadlift training is also commonly employed in rehabilitation programs for individuals with musculoskeletal disorders, thereby contributing to improved quality of life [3].

Athletes often employ different loading schemes at various stages of resistance training to optimize neuromuscular adaptations. In the early phases of training, strength gains are primarily attributed to neural adaptations [4, 5]. Over time, however, factors such as training volume, intensity, rest intervals, and movement velocity become crucial for further strength development [6, 7]. Manipulating these variables through different resistance training models, such as the pyramid and reverse pyramid systems, may optimize outcomes related to strength, hypertrophy, and muscular endurance [8, 9].

Among the various loading paradigms, the pyramid and reverse pyramid methods are widely utilized to promote strength development [10]. Pyramidal loading refers to a progressive resistance training model in which the load increases and the number of repetitions decreases across successive sets, typically starting with lighter weights and higher repetitions. In contrast, reverse pyramidal loading begins with the heaviest load and lowest repetitions in the first set, followed by progressively lighter weights and increased repetitions in subsequent sets, aiming to maximize neural and muscular activation early in the workout [10, 11]. Research supports the efficacy of both approaches. For example, Wade et al. (2014) recommend the pyramid method for maximizing muscle preparation and training to failure [12], whereas Bostani et al. (2012) reported no differences between the effect of pyramid and reverse pyramid methods on muscle strength [13].

Despite growing interest in these methods, their biomechanical implications—particularly on joint coordination—remain poorly understood. The deadlift involves complex intersegmental coordination, especially between the spine and pelvis [14]. Performance improvements and injury prevention may be more closely associated with enhanced coordination and adaptability than with mere increases in force output [15]. The concept of movement variability is central to motor control, recognizing the inherent flexibility and adaptability of the human neuromuscular system [16].

Prior studies have investigated trunk and pelvic coordination during dynamic tasks [14, 17]. However, the choice of biomechanical model for representing the spine is critical to the interpretation of coordination data. Many early investigations treated the spine as a single rigid unit, which obscures the complex, multi-segmental nature of spinal kinematics. Conversely, highly sophisticated modeling approaches exist, including detailed rigid-body lumbar spine models with multiple vertebrae and joints (e.g., de Zee et al., 2007) and specimen-specific finite element models [18]. While these advanced models provide exceptional anatomical and mechanical detail, they are often impractical for dynamic, whole-body tasks such as the deadlift due to computational cost and marker set complexity. A less complex biomechanical model with three segments (pelvis, lumbar spine, and thoracic spine) strikes a balance between fidelity and feasibility for dynamic whole-body tasks such as the deadlift. However, compared with highly detailed multi-segment or vertebra-specific models, this simplified approach may introduce minor limitations in capturing rapid local changes in intersegmental motion due to its reduced segmental resolution. Nevertheless, recent evidence by Grankova et al. (2026) demonstrated good agreement between a clinically compatible marker-based spinal model and more detailed assessments of intersegmental spinal range of motion, supporting the validity of such simplified models for detecting meaningful differences in intersegmental coordination during functional movement tasks [19].

From a functional anatomy perspective, the rationale for separating the upper and lower spinal segments in deadlift analysis is compelling. The lumbar spine, characterized by sagittally-oriented facet joints and greater segmental range of motion, is structurally adapted for large flexion-extension excursions. In contrast, the thoracic spine is constrained by the ribcage and coronally-oriented facets, limiting its sagittal mobility and assigning it a primarily postural stabilization role. Consequently, as the external load increases during the deadlift, we hypothesize that the neuromuscular system will modulate the relative contribution of these anatomically and functionally distinct regions. Specifically, we predict that progressive pyramidal loading will promote greater reliance on distal segments (pelvis, lower lumbar) to generate the requisite hip extensor moment, resulting in a proximal-to-distal shift in segmental dominance. This hypothesis aligns with findings from the rowing literature, where increasing stroke intensity has been shown to alter the distribution of motion between the pelvis and the thoracic spine [20–22]. Although rowing and deadlifting are kinematically distinct, both are bilateral, closed-kinetic-chain activities that require coordinated lumbopelvic force transmission under progressive external load and demand precise intersegmental timing to maintain spinal stability. Therefore, we adopted this three-segment model to test whether load magnitude and loading order (pyramidal vs. reverse pyramidal) differentially influence spine-pelvis coordination strategies during the deadlift, as they do in rowing and gait.

Although previous studies have confirmed the physiological benefits of pyramidal and reverse pyramidal training, the biomechanical mechanisms underlying these outcomes, particularly in terms of joint coordination patterns, have not been adequately examined. Therefore, the present study aimed to compare spine and pelvis coordination variability during deadlifts performed under pyramidal and reverse pyramidal loading conditions. We hypothesized that (1) Spine-pelvis coordination patterns would differ between pyramid and reverse pyramid loading conditions; (2) Coordination variability between spine and pelvis would differ between the two loading schemes.

## Method

### Participants

Twelve trained male powerlifters participated in this study. Their anthropometric characteristics, training history, and strength profiles are summarized as follows (mean ± SD): age = 24.8 ± 3.2 years; height = 178.5 ± 5.4 cm; body mass = 79.6 ± 6.3 kg; BMI = 24.9 ± 1.1 kg/m²; resistance training experience = 5.2 ± 1.8 years; deadlift training frequency = 2.1 ± 0.7 sessions per week. The participants’ mean 10-repetition maximum (10RM) for the conventional deadlift was 118.5 ± 14.2 kg. The estimated one-repetition maximum (1RM), calculated using the Brzycki Eq. (1RM = 10RM × 100 / [102.78–2.78 × 10]), was 137.8 ± 16.5 kg, corresponding to a relative strength ratio of 1.73 ± 0.18 × body mass. The absolute loads corresponding to the three experimental intensities were as follows: 50% 10RM = 59.3 ± 7.1 kg; 70% 10RM = 83.0 ± 9.9 kg; and 90% 10RM = 106.7 ± 12.8 kg. Eligibility criteria included: age between 18 and 30 years, a body mass index (BMI) within the normal to moderately overweight range (20–25 kg/m²), a minimum of two years of consistent resistance training experience, and regular deadlift practice (at least once per week). Participants with a history of low back pain, lower limb injuries, or musculoskeletal surgery within the previous six months were excluded. Informed consent was obtained from all individual participants included in the study. Participants were fully informed about the nature, purpose, and potential risks of the study, and their right to withdraw at any time without consequences was clearly communicated. This study was approved by the Ethics Committee for Human Studies at Kharazmi University (khu-580431). All procedures performed in this study involving human participants were in accordance with the ethical standards of the institutional and/or national research committee and with the 1964 Helsinki Declaration and its later amendments or comparable ethical standards.

### Experimental setup

**Fig. 1:**
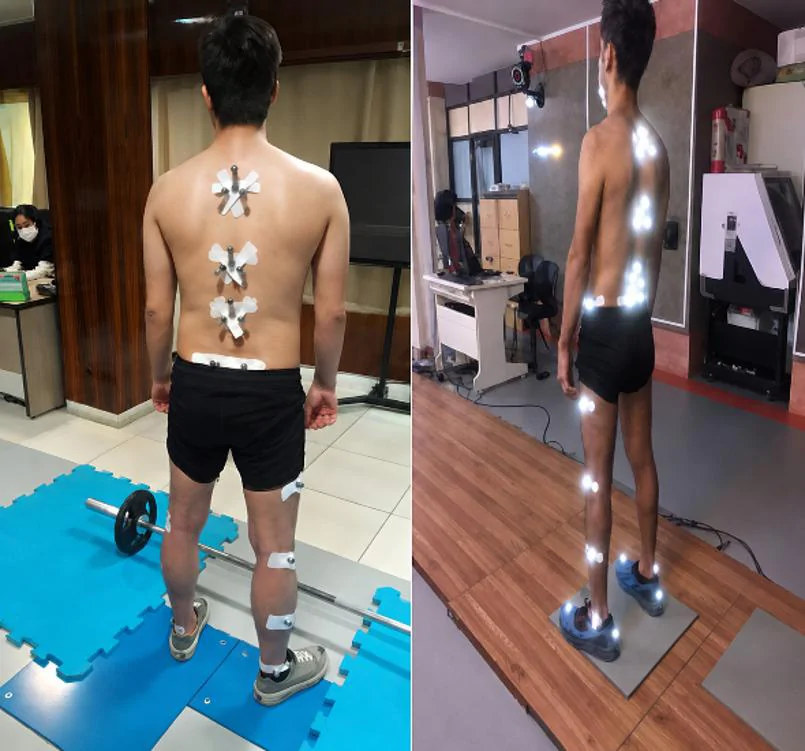
Test execution environment and Location of markers

Three-dimensional kinematic data were recorded using a Vicon Vero 2.2 motion capture system (Oxford Metrics, UK) at a sampling rate of 200 Hz. A lower-limb cluster marker set and a three-segment spinal model were employed to track movement patterns during the deadlift, based on validated biomechanical models [23] (Fig. 1). The barbell was centrally positioned in the testing area, and 48 h prior to testing, each participant’s 10-repetition maximum (10RM) for the deadlift was determined using submaximal testing protocols.

On the test day, participants completed a standardized warm-up that included dynamic movements such as squats and leg swings. The experimental protocol included both static and dynamic trials. Participants were randomly assigned to one of two groups. The first group performed five repetitions of the deadlift using a pyramidal loading pattern (50%, 70%, and 90% of 10RM across sets), while the second group followed a reverse pyramidal pattern using the same relative loads in reverse order. After 48 h, the groups crossed over and performed the alternative loading condition. A two-minute rest period was given between sets. All repetitions were performed using a dead-stop technique, wherein the barbell was lowered under controlled eccentric action, came to a complete rest on the floor for approximately 1–2 s, and was subsequently lifted from a stationary position for the next concentric repetition. This approach was selected to replicate standard powerlifting training protocols and to ensure a consistent mechanical starting state for each repetition. Participants were instructed to perform the lifts at their self-selected natural tempo, replicating their habitual training rhythm, and all lifts were executed using a conventional deadlift technique. To account for inter-individual and inter-condition variability in movement duration, all kinematic data were time-normalized prior to analysis (see Data Processing). 

### Data processing

Kinematic data from five deadlift cycles were extracted per condition. Marker trajectories were labelled, and any gaps resulting from temporary marker occlusion were filled using cubic spline interpolation within Vicon Nexus 2.8.1 (maximum gap length < 100 ms; total gap percentage < 5% of data points across all trials). A zero-phase, fourth-order Butterworth low-pass filter with a 6 Hz cut-off frequency was applied to smooth the data. A three-segment model of the spine (pelvis, lumbar spine, and thoracic spine) was reconstructed using ProCalc software [20, 24].

Movement cycle identification and phase subdivision were both performed using the sagittal-plane knee flexion angle as the sole segmentation criterion. A complete deadlift cycle was defined from the point of maximum knee flexion (start of the concentric phase, with the barbell resting on the floor) to the subsequent point of maximum knee flexion (end of the eccentric phase, with the barbell returned to the floor). Within each cycle, two phases were demarcated: the concentric phase, from maximum knee flexion to minimum knee flexion (full knee extension/lockout), and the eccentric phase, from minimum knee flexion back to the subsequent maximum knee flexion. The knee angle was selected because it provides a direct, unambiguous, and anatomically relevant kinematic landmark that is tightly coupled to the distinct lifting and lowering actions of the deadlift.

To facilitate inter-participant and inter-condition comparisons, each phase was independently time-normalized to 50 data points (0–100% of that phase’s duration) using cubic spline interpolation. Subsequently, the normalized concentric and eccentric time-series were concatenated to form a complete movement cycle of 100 data points, with points 1–50 representing the entire concentric phase and points 51–100 representing the entire eccentric phase. A resolution of 50 points per phase was selected to appropriately preserve the salient kinematic features of each phase given the original sampling characteristics, while maintaining a parsimonious data structure for Statistical Parametric Mapping analysis. It is acknowledged that the established convention in modified vector coding literature is to normalize to 101 points per phase [20, 24]; the 50-point resolution adopted herein represents a pragmatic adaptation suited to the specific duration and sampling parameters of the deadlift task.

### Data analysis

Intersegmental coordination and coordination variability (CV) were quantified using a modified vector coding technique in the sagittal plane [20, 24]. Coordination angles were computed between the following segment pairs: pelvis-lumbar (PL–LB), lumbar–lower thoracic (LB–LT), and lower thoracic–upper thoracic (LT–UT). Four coordination patterns were classified: in-phase with proximal dominance (IPPD), in-phase with distal dominance (IPDD), anti-phase with proximal dominance (APPD), and anti-phase with distal dominance (APDD) [25]. CV was defined as the standard deviation of the coupling angle across time points within each movement cycle. Coordination frequencies were reported to illustrate the prevalence of each pattern across conditions. Time-series plots were generated for coordination variability to visualize differences between loading schemes.

### Statistical analysis

Descriptive statistics (mean ± SD) were computed for all outcome variables. Normality was assessed using the Kolmogorov–Smirnov test. Repeated-measures analysis of variance (ANOVA) was conducted in SPSS v22 (IBM, Chicago, IL, USA) to evaluate differences in coordination patterns across loading conditions and intensities. Statistical Parametric Mapping (SPM) was used in MATLAB (MathWorks, Natick, MA, USA) to compare coordination variability using paired t-tests. The significance level was set at α = 0.05 for all analyses.

## Results

### Coordination patterns

The analysis revealed significant differences in coordination patterns between the pyramidal and reverse pyramidal loading conditions during the deadlift at various intensities and movement phases.

**Fig. 2:**
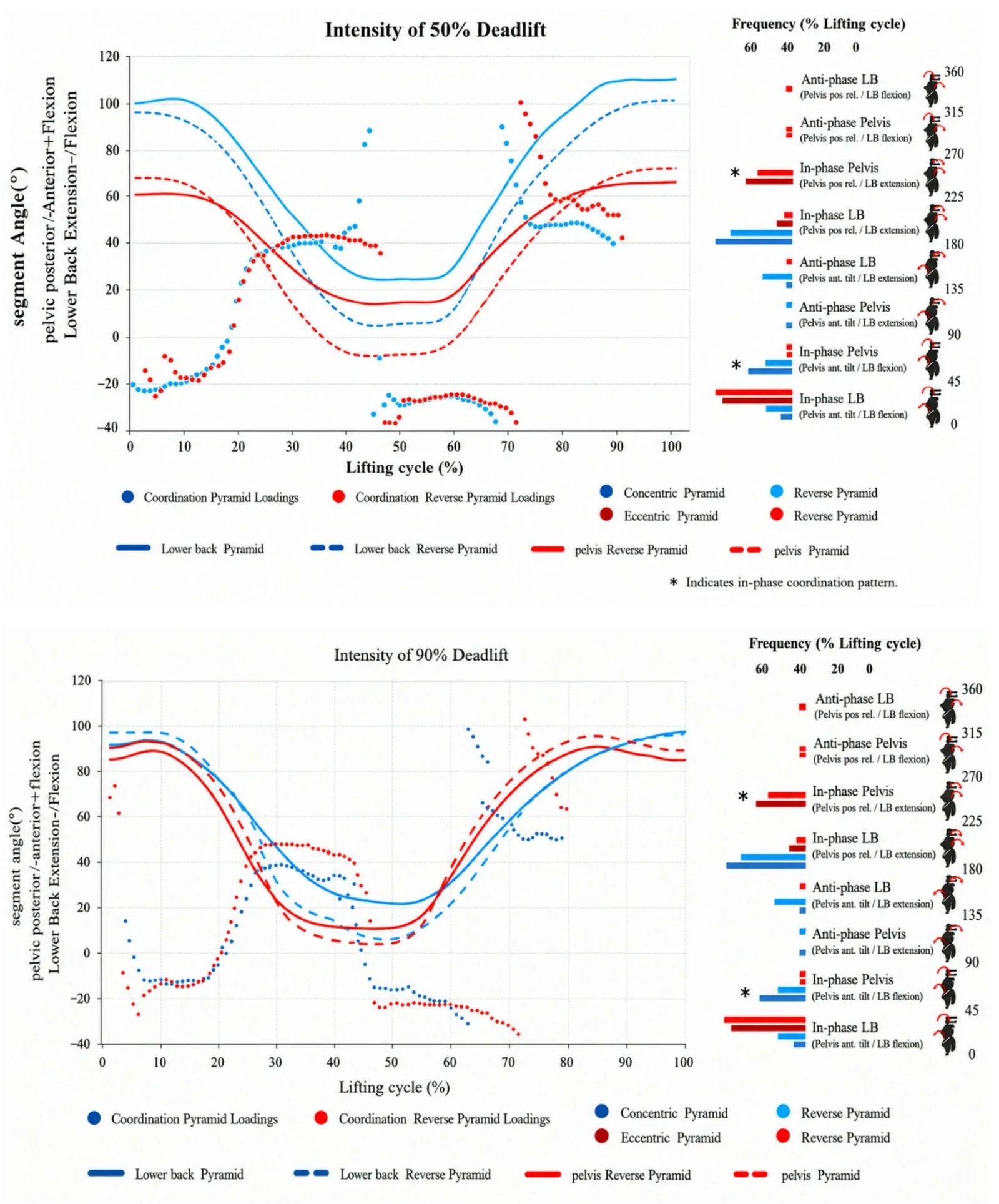
PL/LB segments angular displacement diagram in the sagittal plane and the results of coupling angle frequency

At 50% of 10RM, during the concentric phase (phase 1), the coordination pattern of in-phase with proximal dominance (IPPD) in the PL–LB segment was significantly reduced under pyramidal loading compared to reverse pyramidal loading (*p* = 0.018). At 90% of 10RM, both the concentric (phase 1) and eccentric (phase 2) phases showed a significant increase in in-phase with distal dominance (IPDD) in the PL–LB segment under pyramidal loading compared to the reverse pyramidal condition (*p* = 0.039 and *p* = 0.043, respectively) (Fig. 2).

**Fig. 3:**
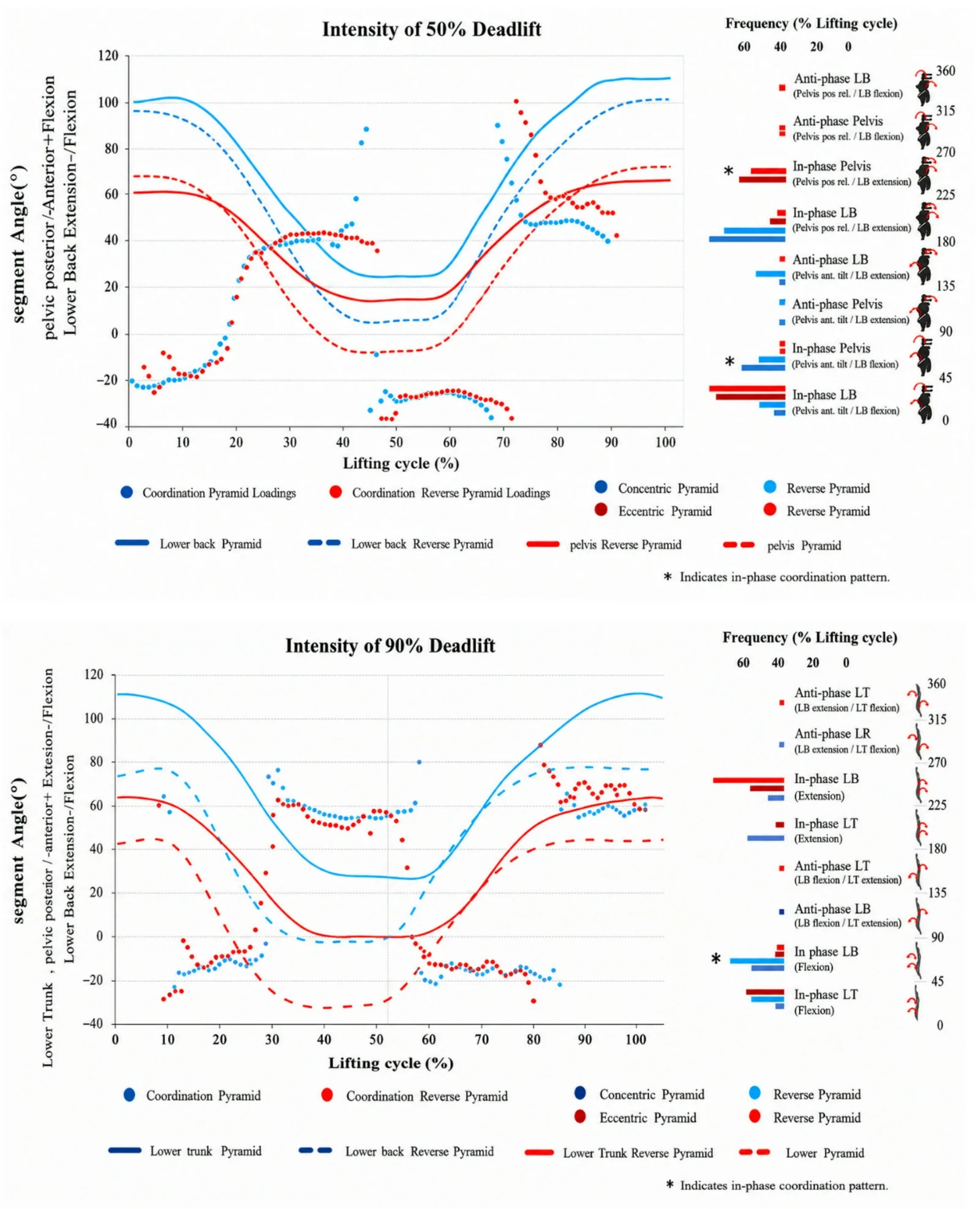
LB_LT segments angular displacement diagram in the sagittal plane and the results of coupling angle frequency

In the LB–LT segment, under 50% loading during the concentric phase, the anti-phase with distal dominance (APDD) pattern was significantly lower in the pyramidal condition compared to the reverse pyramidal condition (*p* = 0.045). However, at 90% intensity, the anti-phase with proximal dominance (APPD) pattern significantly increased in the pyramidal condition (*p* = 0.037) (Fig. 3).

**Fig. 4:**
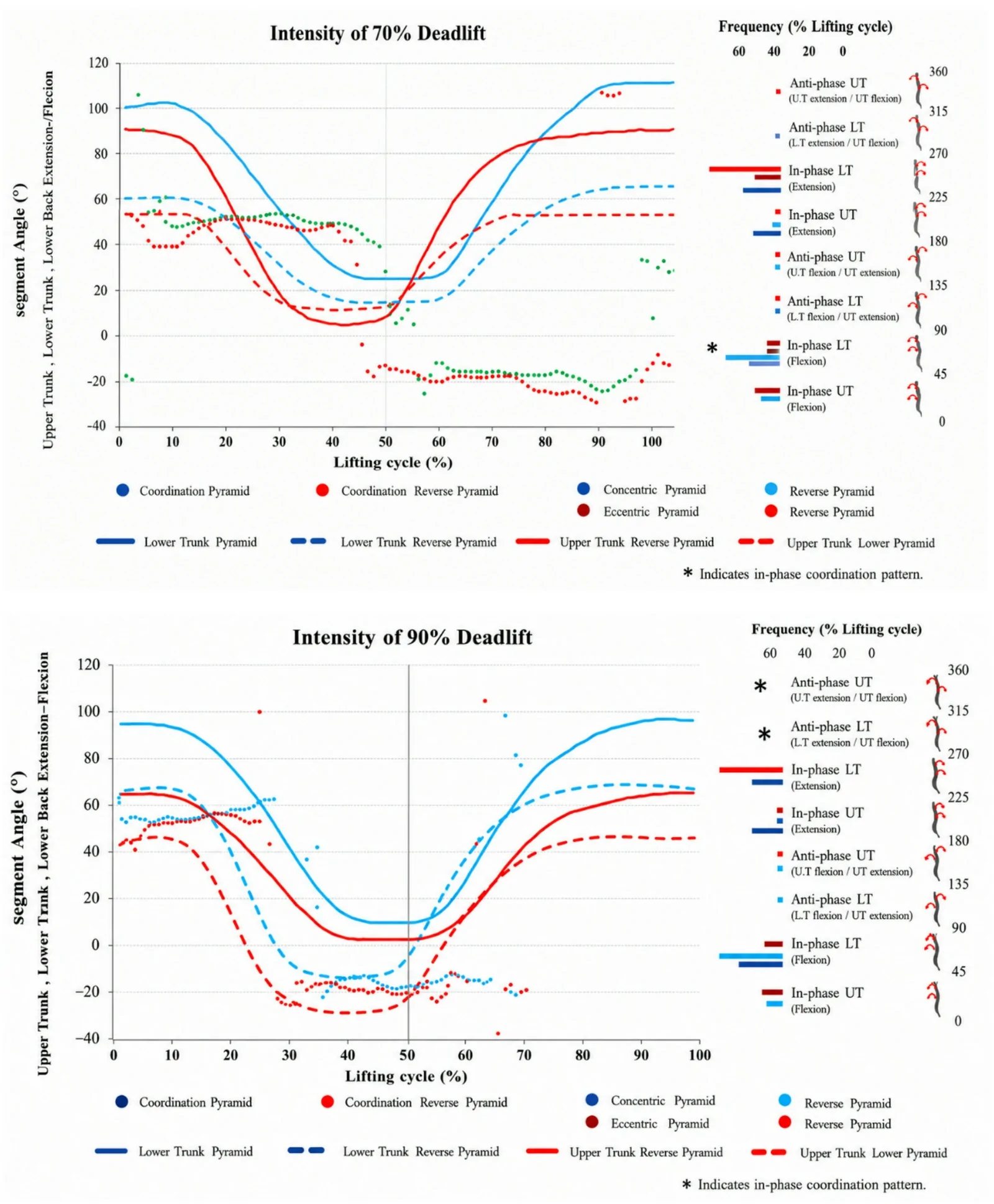
LT_UT segments angular displacement diagram in the sagittal plane and the results of coupling angle frequency

In the LT–UT segment, at 70% of 10RM during the concentric phase, the in-phase with distal dominance (IPDD) pattern was significantly higher under pyramidal loading (*p* = 0.029). At 90% intensity, the frequency of both APDD and APPD patterns significantly decreased in the pyramidal condition (*p* = 0.030 and *p* = 0.035, respectively) (Fig. 4). No additional significant differences were observed in coordination patterns across segments and phases beyond the aforementioned cases.

### Coordination variability

The statistical parametric mapping (SPM) analysis using repeated-measures t-tests showed no statistically significant differences in the variability of coordination patterns between the pyramidal and reverse pyramidal loading conditions (*p* > 0.05). This finding indicates that, despite differences in coordination pattern frequencies, the overall coordination variability across the sagittal plane was not significantly influenced by the type of loading protocol used.

## Discussion

This study investigated the effects of pyramidal and reverse pyramidal loading schemes on spine and pelvis coordination and its variability during the deadlift in trained male athletes. The findings partially confirmed the initial hypotheses: significant differences in intersegmental coordination patterns were observed across different loading intensities and movement phases, whereas coordination variability remained statistically unchanged between the two loading methods. These outcomes provide meaningful insight into how different resistance training strategies may differentially affect movement coordination across the lumbopelvic and thoracic regions during high-load exercises such as the deadlift.

## Coordination patterns and segmental dominance

The deadlift requires integrated multi-joint control, particularly through the pelvis and spinal segments. Our findings demonstrated that under pyramidal loading, which involves a progressive increase in load, distal segments (e.g., pelvis and lower thoracic spine) demonstrated greater dominance, particularly at high intensities (90% 10RM). This increased distal influence was observed in the PL–LB and LT–UT couplings and may reflect adaptive load-mediated strategies to manage load through greater momentum and pelvic drive in the concentric phase [14, 26]. This observation is consistent with Fadaei et al. (2021), who showed that in trained individuals, in-phase coupling with pelvic or lower lumbar dominance is common during heavy lifting, particularly in athletes with or at risk of chronic low back pain [27]. However, our findings add to this by showing that loading structure—not just magnitude—influences dominance, with pyramidal loading promoting earlier distal engagement. This may lead to greater mechanical demand on the sacroiliac joint and lower lumbar vertebrae, thereby increasing cumulative tissue stress during repetitive training.

Additionally, the transition from anti-phase to in-phase coordination in the LB–LT segment as load increased is indicative of reduced kinematic independence between the lumbar and lower thoracic segments. This shift toward tighter intersegmental coupling is consistent with Bernstein’s (1967) principle of ‘freezing’ mechanical degrees of freedom—a well-established motor control strategy in which the central nervous system simplifies the coordination of multiple segments by constraining their independent motion when task demands increase. Rather than representing a loss of motor control, this reorganization reflects an adaptive strategy that prioritizes spinal stability and mechanical efficiency under high external load [28].

The shift to anti-phase patterns in the LT–UT coupling at 90% intensity in pyramidal loading suggests that as load increases, recruitment of thoracic motion becomes necessary to maintain trunk alignment. This mirrors observations by Leardini et al. (2011), who found that segmental decoupling in the thoracic spine emerges under fatigue or mechanical stress [21]. In our case, this may represent a postural correction strategy, in which the upper trunk resists further flexion as the lower thoracic segment extends. In summary, the findings suggest that pyramidal loading, due to its progressive overload, challenges intersegmental control mechanisms more than reverse pyramidal loading, which starts heavy but quickly deloads. These mechanical adaptations may have implications for injury risk, particularly in settings involving repetitive heavy lifts with incomplete recovery.

### Coordination variability and motor control

Contrary to our second hypothesis, no statistically significant differences were observed in coordination variability between loading schemes. While prior literature associates greater variability with functional adaptability [29, 30], especially in novice populations, it is possible that the trained athletes in our study had already optimized their motor control strategies and thus maintained consistent variability across conditions. This interpretation is supported by Lamoth et al. (2006), who showed that repetitive training reduces unnecessary variability and refines intersegmental control, particularly in tasks involving the trunk [17].

Moreover, Needham et al. (2020) emphasized that vector coding analysis of spinal coordination can reveal subtle changes in segmental control not evident through standard joint kinematics [31]. While we observed coordination pattern shifts, the stability of variability may indicate robust adaptive mechanisms in trained individuals. This further highlights the importance of considering both pattern and variability metrics together when analyzing trunk dynamics under load. However, it must be noted that the range of load intensities tested (50%, 70%, 90%) and the limited number of repetitions per condition may not have been sufficient to induce neuromuscular fatigue or perturbatory responses that elicit greater variability shifts. Future studies may benefit from incorporating longer sets, fatigue protocols, or perturbation-based deadlifts to explore the dynamic limits of coordination adaptability.

### Implications for practice

The results of this study have practical implications for strength and conditioning professionals, particularly in prescribing deadlift variations for trained, asymptomatic athletes. While both pyramidal and reverse pyramidal schemes are effective for strength development [10], the pyramidal model may impose greater mechanical and neuromuscular challenges on the spine and pelvis, particularly under high loads. Coaches should consider these findings when designing training blocks, especially for athletes in high-volume training phases or those returning to training following a period of deconditioning. Additionally, the emergence of segment-specific dominance patterns under load suggests that targeted mobility and stability training (e.g., hip control, lumbar-pelvic rhythm drills) may help reduce adaptive movement patterns and promote more balanced spine loading. However, it is critical to emphasize that these findings were obtained from healthy, well-trained powerlifters and cannot be directly generalized to clinical populations, including individuals with acute or chronic low back pain. The neuromuscular strategies, pain-related adaptations, and tissue loading tolerances in patient populations may differ substantially from those observed in asymptomatic trained individuals. Therefore, while the coordination metrics employed in this study may offer a promising framework for future clinical biomechanics research, their application to rehabilitation settings requires dedicated investigation in patient cohorts.

## Conclusion

The findings of this study provide valuable insight into the biomechanical effects of pyramidal and reverse pyramidal loading on spine and pelvis coordination during the deadlift. Across all tested intensities (50%, 70%, and 90% of 10RM), the in-phase coordination pattern was consistently dominant in the pelvis–lumbar (PL–LB) and lumbar–lower thoracic (LB–LT) segment couplings. However, under pyramidal loading, particularly in the sagittal plane, a disruption in the transmission of movement from distal to proximal segments was observed. This impaired intersegmental coordination was associated with greater mechanical demand on the distal couplings, potentially increasing the risk of overuse injuries and localized pain. Notably, these effects were most evident in the PL–LB coupling across both movement phases, and in the LB–LT and lower thoracic–upper thoracic (LT–UT) couplings during the concentric phase of pyramidal loading.

These results suggest that while both loading models are effective, pyramidal loading may impose greater neuromechanical demands on the lower trunk segments. Therefore, coaches and athletes are advised to apply pyramidal loading schemes with caution, particularly under high-load conditions, and may consider using reverse pyramidal loading as a safer alternative to reduce mechanical stress during resistance training. Importantly, no significant differences in coordination variability were found between the two loading strategies. This may reflect the stable movement strategies employed by trained lifters. However, given that the study examined only three submaximal intensities (50%, 70%, and 90% of 10RM), it remains possible that different outcomes would be observed under higher intensities such as 1RM or fatigue-inducing protocols. Future research should investigate a broader range of loading conditions—including maximal and fatigue-inducing protocols—and incorporate additional variables such as muscle activity, joint kinetics, and perceived exertion. In particular, extending this line of inquiry to clinical populations with low back pain or utilizing experimental pain models (e.g., hypertonic saline injection) would help determine whether the coordination strategies observed in asymptomatic lifters are altered under painful conditions. Furthermore, the application of inertial measurement unit (IMU)-based musculoskeletal models (e.g., Sibson et al., 2024) may enable the simultaneous assessment of spinal kinematics and compressive loading in ecologically valid field settings, overcoming the laboratory constraints inherent to marker-based motion capture [32]. Such integrated approaches would substantially advance our understanding of how loading prescription modulates both the biomechanical demands and the neuromuscular control of the spine during resistance training.

Additionally, while participants performed the deadlift at their self-selected tempo to preserve ecological validity, formal measurement and statistical comparison of movement duration between conditions were not conducted. Although time-normalization of kinematic data mitigates the direct effect of tempo on coordination metrics, future studies may wish to quantify and control for lifting cadence as a potential covariate.

List of figures.

## Data Availability

The datasets generated and/or analyzed during the current study are available from the corresponding author on reasonable request.

## Abbreviations

10RM: Ten-repetition maximum
1RM: One-repetition maximum
PL–LB: Pelvis–Lumbar spine
LB–LT: Lumbar spine–Lower thoracic spine
LT–UT: Lower thoracic spine–Upper thoracic spine
IPPD: In-phase with proximal dominance
IPDD: In-phase with distal dominance
APPD: Anti-phase with proximal dominance
APDD: Anti-phase with distal dominance
CV: Coordination variability
SPM: Statistical Parametric Mapping
BMI: Body Mass Index
